# Supplementary material for: Resolving the full spectrum of human genome variation using Linked-Reads
Source: Genome Res. 2019 Apr;29(4):635–45. doi: 10.1101/gr.234443.118 (PMC6442396; doi:10.1101/gr.234443.118)
Supplement: Supplemental Material [file supp_gr.234443.118_Supplemental_longranger-2.2.zip › longranger-2.2.tar.bz2!/lr2.2-public/lib/stan/cmdstan/stan/doc/api/html/inv__gamma_8hpp_source.html]

Stan: src/stan/prob/distributions/univariate/continuous/inv\_gamma.hpp Source File


|  |  |
| --- | --- |
|  | Stan  2.5.0  probability, sampling & optimization |


- Main Page
- Related Pages
- Namespaces
- Classes
- Files

- File List
- File Members

All Classes Namespaces Files Functions Variables Typedefs Enumerations Enumerator Friends Macros Pages

- src
- stan
- prob
- distributions
- univariate
- continuous

inv\_gamma.hpp

Go to the documentation of this file.

1 #ifndef STAN\_\_PROB\_\_DISTRIBUTIONS\_\_UNIVARIATE\_\_CONTINUOUS\_\_INV\_GAMMA\_HPP

2 #define STAN\_\_PROB\_\_DISTRIBUTIONS\_\_UNIVARIATE\_\_CONTINUOUS\_\_INV\_GAMMA\_HPP

3

4 #include <boost/random/gamma\_distribution.hpp>

5 #include <boost/random/variate\_generator.hpp>

6

7 #include <stan/agrad/partials\_vari.hpp>

8 #include <stan/math/error\_handling.hpp>

9 #include <stan/math/constants.hpp>

10 #include <stan/math/functions/multiply\_log.hpp>

11 #include <stan/math/functions/value\_of.hpp>

12 #include <stan/meta/traits.hpp>

13 #include <stan/prob/constants.hpp>

14 #include <stan/prob/traits.hpp>

15 #include <stan/prob/internal\_math.hpp>

16

17 namespace stan {

18

19  namespace prob {

20

37  template <bool propto,

38  typename T\_y, typename T\_shape, typename T\_scale>

39  typename return\_type<T\_y,T\_shape,T\_scale>::type

40  inv\_gamma\_log(const T\_y& y, const T\_shape& alpha, const T\_scale& beta) {

41  static const char\* function = "stan::prob::inv\_gamma\_log(%1%)";

42

43  using stan::is\_constant\_struct;

44  using stan::math::check\_not\_nan;

45  using stan::math::check\_positive\_finite;

46  using boost::math::tools::promote\_args;

47  using stan::math::check\_consistent\_sizes;

48  using stan::math::value\_of;

49

50  // check if any vectors are zero length

51  if (!(stan::length(y)

52  && stan::length(alpha)

53  && stan::length(beta)))

54  return 0.0;

55

56  // set up return value accumulator

57  double logp(0.0);

58

59  check\_not\_nan(function, y, "Random variable", &logp);

60  check\_positive\_finite(function, alpha, "Shape parameter", &logp);

61  check\_positive\_finite(function, beta, "Scale parameter", &logp);

62  check\_consistent\_sizes(function,

63  y,alpha,beta,

64  "Random variable","Shape parameter",

65  "Scale parameter",

66  &logp);

67

68  // check if no variables are involved and prop-to

69  if (!include\_summand<propto,T\_y,T\_shape,T\_scale>::value)

70  return 0.0;

71

72  // set up template expressions wrapping scalars into vector views

73  VectorView<const T\_y> y\_vec(y);

74  VectorView<const T\_shape> alpha\_vec(alpha);

75  VectorView<const T\_scale> beta\_vec(beta);

76

77  for (size\_t n = 0; n < length(y); n++) {

78  const double y\_dbl = value\_of(y\_vec[n]);

79  if (y\_dbl <= 0)

80  return LOG\_ZERO;

81  }

82

83  size\_t N = max\_size(y, alpha, beta);

84  agrad::OperandsAndPartials<T\_y, T\_shape, T\_scale>

85  operands\_and\_partials(y, alpha, beta);

86

87  using boost::math::lgamma;

88  using stan::math::multiply\_log;

89  using boost::math::digamma;

90

91  DoubleVectorView<include\_summand<propto,T\_y,T\_shape>::value,

92  is\_vector<T\_y>::value>

93  log\_y(length(y));

94  DoubleVectorView<include\_summand<propto,T\_y,T\_scale>::value,

95  is\_vector<T\_y>::value>

96  inv\_y(length(y));

97  for(size\_t n = 0; n < length(y); n++) {

98  if (include\_summand<propto,T\_y,T\_shape>::value)

99  if (value\_of(y\_vec[n]) > 0)

100  log\_y[n] = log(value\_of(y\_vec[n]));

101  if (include\_summand<propto,T\_y,T\_scale>::value)

102  inv\_y[n] = 1.0 / value\_of(y\_vec[n]);

103  }

104

105  DoubleVectorView<include\_summand<propto,T\_shape>::value,

106  is\_vector<T\_shape>::value>

107  lgamma\_alpha(length(alpha));

108  DoubleVectorView<!is\_constant\_struct<T\_shape>::value,

109  is\_vector<T\_shape>::value>

110  digamma\_alpha(length(alpha));

111  for (size\_t n = 0; n < length(alpha); n++) {

112  if (include\_summand<propto,T\_shape>::value)

113  lgamma\_alpha[n] = lgamma(value\_of(alpha\_vec[n]));

114  if (!is\_constant\_struct<T\_shape>::value)

115  digamma\_alpha[n] = digamma(value\_of(alpha\_vec[n]));

116  }

117

118  DoubleVectorView<include\_summand<propto,T\_shape,T\_scale>::value,

119  is\_vector<T\_scale>::value>

120  log\_beta(length(beta));

121  if (include\_summand<propto,T\_shape,T\_scale>::value)

122  for (size\_t n = 0; n < length(beta); n++)

123  log\_beta[n] = log(value\_of(beta\_vec[n]));

124

125  for (size\_t n = 0; n < N; n++) {

126  // pull out values of arguments

127  const double alpha\_dbl = value\_of(alpha\_vec[n]);

128  const double beta\_dbl = value\_of(beta\_vec[n]);

129

130  if (include\_summand<propto,T\_shape>::value)

131  logp -= lgamma\_alpha[n];

132  if (include\_summand<propto,T\_shape,T\_scale>::value)

133  logp += alpha\_dbl \* log\_beta[n];

134  if (include\_summand<propto,T\_y,T\_shape>::value)

135  logp -= (alpha\_dbl+1.0) \* log\_y[n];

136  if (include\_summand<propto,T\_y,T\_scale>::value)

137  logp -= beta\_dbl \* inv\_y[n];

138

139  // gradients

140  if (!is\_constant<typename is\_vector<T\_y>::type>::value)

141  operands\_and\_partials.d\_x1[n]

142  += -(alpha\_dbl+1) \* inv\_y[n] + beta\_dbl \* inv\_y[n] \* inv\_y[n];

143  if (!is\_constant<typename is\_vector<T\_shape>::type>::value)

144  operands\_and\_partials.d\_x2[n]

145  += -digamma\_alpha[n] + log\_beta[n] - log\_y[n];

146  if (!is\_constant<typename is\_vector<T\_scale>::type>::value)

147  operands\_and\_partials.d\_x3[n] += alpha\_dbl / beta\_dbl - inv\_y[n];

148  }

149  return operands\_and\_partials.to\_var(logp);

150  }

151

152  template <typename T\_y, typename T\_shape, typename T\_scale>

153  inline

154  typename return\_type<T\_y,T\_shape,T\_scale>::type

155  inv\_gamma\_log(const T\_y& y, const T\_shape& alpha, const T\_scale& beta) {

156  return inv\_gamma\_log<false>(y,alpha,beta);

157  }

158

175  template <typename T\_y, typename T\_shape, typename T\_scale>

176  typename return\_type<T\_y,T\_shape,T\_scale>::type

177  inv\_gamma\_cdf(const T\_y& y, const T\_shape& alpha, const T\_scale& beta) {

178

179  // Size checks

180  if (!(stan::length(y) && stan::length(alpha) && stan::length(beta)))

181  return 1.0;

182

183  // Error checks

184  static const char\* function = "stan::prob::inv\_gamma\_cdf(%1%)";

185

186  using stan::math::check\_positive\_finite;

187  using stan::math::check\_not\_nan;

188  using stan::math::check\_consistent\_sizes;

189  using stan::math::check\_greater\_or\_equal;

190  using stan::math::check\_less\_or\_equal;

191  using stan::math::check\_nonnegative;

192  using stan::math::value\_of;

193  using boost::math::tools::promote\_args;

194

195  double P(1.0);

196

197  check\_positive\_finite(function, alpha, "Shape parameter", &P);

198  check\_positive\_finite(function, beta, "Scale parameter", &P);

199  check\_not\_nan(function, y, "Random variable", &P);

200  check\_nonnegative(function, y, "Random variable", &P);

201  check\_consistent\_sizes(function, y, alpha, beta,

202  "Random variable", "Shape parameter",

203  "Scale Parameter",

204  &P);

205

206  // Wrap arguments in vectors

207  VectorView<const T\_y> y\_vec(y);

208  VectorView<const T\_shape> alpha\_vec(alpha);

209  VectorView<const T\_scale> beta\_vec(beta);

210  size\_t N = max\_size(y, alpha, beta);

211

212  agrad::OperandsAndPartials<T\_y, T\_shape, T\_scale>

213  operands\_and\_partials(y, alpha, beta);

214

215  // Explicit return for extreme values

216  // The gradients are technically ill-defined, but treated as zero

217

218  for (size\_t i = 0; i < stan::length(y); i++) {

219  if (value\_of(y\_vec[i]) == 0)

220  return operands\_and\_partials.to\_var(0.0);

221  }

222

223  // Compute CDF and its gradients

224  using boost::math::gamma\_p\_derivative;

225  using boost::math::gamma\_q;

226  using boost::math::digamma;

227  using boost::math::tgamma;

228

229  // Cache a few expensive function calls if nu is a parameter

230  DoubleVectorView<!is\_constant\_struct<T\_shape>::value,

231  is\_vector<T\_shape>::value>

232  gamma\_vec(stan::length(alpha));

233  DoubleVectorView<!is\_constant\_struct<T\_shape>::value,

234  is\_vector<T\_shape>::value>

235  digamma\_vec(stan::length(alpha));

236

237  if (!is\_constant\_struct<T\_shape>::value) {

238  for (size\_t i = 0; i < stan::length(alpha); i++) {

239  const double alpha\_dbl = value\_of(alpha\_vec[i]);

240  gamma\_vec[i] = tgamma(alpha\_dbl);

241  digamma\_vec[i] = digamma(alpha\_dbl);

242  }

243  }

244

245  // Compute vectorized CDF and gradient

246  for (size\_t n = 0; n < N; n++) {

247  // Explicit results for extreme values

248  // The gradients are technically ill-defined, but treated as zero

249  if (value\_of(y\_vec[n]) == std::numeric\_limits<double>::infinity())

250  continue;

251

252  // Pull out values

253  const double y\_dbl = value\_of(y\_vec[n]);

254  const double y\_inv\_dbl = 1.0 / y\_dbl;

255  const double alpha\_dbl = value\_of(alpha\_vec[n]);

256  const double beta\_dbl = value\_of(beta\_vec[n]);

257

258  // Compute

259  const double Pn = gamma\_q(alpha\_dbl, beta\_dbl \* y\_inv\_dbl);

260

261  P \*= Pn;

262

263  if (!is\_constant\_struct<T\_y>::value)

264  operands\_and\_partials.d\_x1[n]

265  += beta\_dbl \* y\_inv\_dbl \* y\_inv\_dbl

266  \* gamma\_p\_derivative(alpha\_dbl, beta\_dbl \* y\_inv\_dbl)

267  / Pn;

268  if (!is\_constant\_struct<T\_shape>::value)

269  operands\_and\_partials.d\_x2[n]

270  += stan::math::gradRegIncGamma(alpha\_dbl, beta\_dbl

271  \* y\_inv\_dbl, gamma\_vec[n],

272  digamma\_vec[n]) / Pn;

273  if (!is\_constant\_struct<T\_scale>::value)

274  operands\_and\_partials.d\_x3[n]

275  += - y\_inv\_dbl \* gamma\_p\_derivative(alpha\_dbl,

276  beta\_dbl \* y\_inv\_dbl) / Pn;

277  }

278

279  if (!is\_constant\_struct<T\_y>::value)

280  for (size\_t n = 0; n < stan::length(y); ++n)

281  operands\_and\_partials.d\_x1[n] \*= P;

282  if (!is\_constant\_struct<T\_shape>::value)

283  for (size\_t n = 0; n < stan::length(alpha); ++n)

284  operands\_and\_partials.d\_x2[n] \*= P;

285  if (!is\_constant\_struct<T\_scale>::value)

286  for (size\_t n = 0; n < stan::length(beta); ++n)

287  operands\_and\_partials.d\_x3[n] \*= P;

288

289  return operands\_and\_partials.to\_var(P);

290  }

291

292  template <typename T\_y, typename T\_shape, typename T\_scale>

293  typename return\_type<T\_y,T\_shape,T\_scale>::type

294  inv\_gamma\_cdf\_log(const T\_y& y, const T\_shape& alpha, const T\_scale& beta) {

295

296  // Size checks

297  if (!(stan::length(y) && stan::length(alpha) && stan::length(beta)))

298  return 0.0;

299

300  // Error checks

301  static const char\* function = "stan::prob::inv\_gamma\_cdf\_log(%1%)";

302

303  using stan::math::check\_positive\_finite;

304  using stan::math::check\_not\_nan;

305  using stan::math::check\_consistent\_sizes;

306  using stan::math::check\_greater\_or\_equal;

307  using stan::math::check\_less\_or\_equal;

308  using stan::math::check\_nonnegative;

309  using stan::math::value\_of;

310  using boost::math::tools::promote\_args;

311

312  double P(0.0);

313

314  check\_positive\_finite(function, alpha, "Shape parameter", &P);

315  check\_positive\_finite(function, beta, "Scale parameter", &P);

316  check\_not\_nan(function, y, "Random variable", &P);

317  check\_nonnegative(function, y, "Random variable", &P);

318  check\_consistent\_sizes(function, y, alpha, beta,

319  "Random variable", "Shape parameter",

320  "Scale Parameter",

321  &P);

322

323  // Wrap arguments in vectors

324  VectorView<const T\_y> y\_vec(y);

325  VectorView<const T\_shape> alpha\_vec(alpha);

326  VectorView<const T\_scale> beta\_vec(beta);

327  size\_t N = max\_size(y, alpha, beta);

328

329  agrad::OperandsAndPartials<T\_y, T\_shape, T\_scale>

330  operands\_and\_partials(y, alpha, beta);

331

332  // Explicit return for extreme values

333  // The gradients are technically ill-defined, but treated as zero

334

335  for (size\_t i = 0; i < stan::length(y); i++) {

336  if (value\_of(y\_vec[i]) == 0)

337  return operands\_and\_partials.to\_var(stan::math::negative\_infinity());

338  }

339

340  // Compute cdf\_log and its gradients

341  using boost::math::gamma\_p\_derivative;

342  using boost::math::gamma\_q;

343  using boost::math::digamma;

344  using boost::math::tgamma;

345

346  // Cache a few expensive function calls if nu is a parameter

347  DoubleVectorView<!is\_constant\_struct<T\_shape>::value,

348  is\_vector<T\_shape>::value>

349  gamma\_vec(stan::length(alpha));

350  DoubleVectorView<!is\_constant\_struct<T\_shape>::value,

351  is\_vector<T\_shape>::value>

352  digamma\_vec(stan::length(alpha));

353

354  if (!is\_constant\_struct<T\_shape>::value) {

355  for (size\_t i = 0; i < stan::length(alpha); i++) {

356  const double alpha\_dbl = value\_of(alpha\_vec[i]);

357  gamma\_vec[i] = tgamma(alpha\_dbl);

358  digamma\_vec[i] = digamma(alpha\_dbl);

359  }

360  }

361

362  // Compute vectorized cdf\_log and gradient

363  for (size\_t n = 0; n < N; n++) {

364  // Explicit results for extreme values

365  // The gradients are technically ill-defined, but treated as zero

366  if (value\_of(y\_vec[n]) == std::numeric\_limits<double>::infinity())

367  continue;

368

369  // Pull out values

370  const double y\_dbl = value\_of(y\_vec[n]);

371  const double y\_inv\_dbl = 1.0 / y\_dbl;

372  const double alpha\_dbl = value\_of(alpha\_vec[n]);

373  const double beta\_dbl = value\_of(beta\_vec[n]);

374

375  // Compute

376  const double Pn = gamma\_q(alpha\_dbl, beta\_dbl \* y\_inv\_dbl);

377

378  P += log(Pn);

379

380  if (!is\_constant\_struct<T\_y>::value)

381  operands\_and\_partials.d\_x1[n]

382  += beta\_dbl \* y\_inv\_dbl \* y\_inv\_dbl

383  \* gamma\_p\_derivative(alpha\_dbl, beta\_dbl \* y\_inv\_dbl)

384  / Pn;

385  if (!is\_constant\_struct<T\_shape>::value)

386  operands\_and\_partials.d\_x2[n]

387  += stan::math::gradRegIncGamma(alpha\_dbl, beta\_dbl

388  \* y\_inv\_dbl, gamma\_vec[n],

389  digamma\_vec[n]) / Pn;

390  if (!is\_constant\_struct<T\_scale>::value)

391  operands\_and\_partials.d\_x3[n]

392  += - y\_inv\_dbl \* gamma\_p\_derivative(alpha\_dbl,

393  beta\_dbl \* y\_inv\_dbl) / Pn;

394  }

395

396  return operands\_and\_partials.to\_var(P);

397  }

398

399  template <typename T\_y, typename T\_shape, typename T\_scale>

400  typename return\_type<T\_y,T\_shape,T\_scale>::type

401  inv\_gamma\_ccdf\_log(const T\_y& y, const T\_shape& alpha, const T\_scale& beta) {

402

403  // Size checks

404  if (!(stan::length(y) && stan::length(alpha) && stan::length(beta)))

405  return 0.0;

406

407  // Error checks

408  static const char\* function = "stan::prob::inv\_gamma\_ccdf\_log(%1%)";

409

410  using stan::math::check\_positive\_finite;

411  using stan::math::check\_not\_nan;

412  using stan::math::check\_consistent\_sizes;

413  using stan::math::check\_greater\_or\_equal;

414  using stan::math::check\_less\_or\_equal;

415  using stan::math::check\_nonnegative;

416  using stan::math::value\_of;

417  using boost::math::tools::promote\_args;

418

419  double P(0.0);

420

421  check\_positive\_finite(function, alpha, "Shape parameter", &P);

422  check\_positive\_finite(function, beta, "Scale parameter", &P);

423  check\_not\_nan(function, y, "Random variable", &P);

424  check\_nonnegative(function, y, "Random variable", &P);

425  check\_consistent\_sizes(function, y, alpha, beta,

426  "Random variable", "Shape parameter",

427  "Scale Parameter",

428  &P);

429

430  // Wrap arguments in vectors

431  VectorView<const T\_y> y\_vec(y);

432  VectorView<const T\_shape> alpha\_vec(alpha);

433  VectorView<const T\_scale> beta\_vec(beta);

434  size\_t N = max\_size(y, alpha, beta);

435

436  agrad::OperandsAndPartials<T\_y, T\_shape, T\_scale>

437  operands\_and\_partials(y, alpha, beta);

438

439  // Explicit return for extreme values

440  // The gradients are technically ill-defined, but treated as zero

441

442  for (size\_t i = 0; i < stan::length(y); i++) {

443  if (value\_of(y\_vec[i]) == 0)

444  return operands\_and\_partials.to\_var(0.0);

445  }

446

447  // Compute ccdf\_log and its gradients

448  using boost::math::gamma\_p\_derivative;

449  using boost::math::gamma\_q;

450  using boost::math::digamma;

451  using boost::math::tgamma;

452

453  // Cache a few expensive function calls if nu is a parameter

454  DoubleVectorView<!is\_constant\_struct<T\_shape>::value,

455  is\_vector<T\_shape>::value>

456  gamma\_vec(stan::length(alpha));

457  DoubleVectorView<!is\_constant\_struct<T\_shape>::value,

458  is\_vector<T\_shape>::value>

459  digamma\_vec(stan::length(alpha));

460

461  if (!is\_constant\_struct<T\_shape>::value) {

462  for (size\_t i = 0; i < stan::length(alpha); i++) {

463  const double alpha\_dbl = value\_of(alpha\_vec[i]);

464  gamma\_vec[i] = tgamma(alpha\_dbl);

465  digamma\_vec[i] = digamma(alpha\_dbl);

466  }

467  }

468

469  // Compute vectorized ccdf\_log and gradient

470  for (size\_t n = 0; n < N; n++) {

471  // Explicit results for extreme values

472  // The gradients are technically ill-defined, but treated as zero

473  if (value\_of(y\_vec[n]) == std::numeric\_limits<double>::infinity())

474  return operands\_and\_partials.to\_var(stan::math::negative\_infinity());

475

476  // Pull out values

477  const double y\_dbl = value\_of(y\_vec[n]);

478  const double y\_inv\_dbl = 1.0 / y\_dbl;

479  const double alpha\_dbl = value\_of(alpha\_vec[n]);

480  const double beta\_dbl = value\_of(beta\_vec[n]);

481

482  // Compute

483  const double Pn = 1.0 - gamma\_q(alpha\_dbl, beta\_dbl \* y\_inv\_dbl);

484

485  P += log(Pn);

486

487  if (!is\_constant\_struct<T\_y>::value)

488  operands\_and\_partials.d\_x1[n]

489  -= beta\_dbl \* y\_inv\_dbl \* y\_inv\_dbl

490  \* gamma\_p\_derivative(alpha\_dbl, beta\_dbl \* y\_inv\_dbl)

491  / Pn;

492  if (!is\_constant\_struct<T\_shape>::value)

493  operands\_and\_partials.d\_x2[n]

494  -= stan::math::gradRegIncGamma(alpha\_dbl, beta\_dbl

495  \* y\_inv\_dbl, gamma\_vec[n],

496  digamma\_vec[n]) / Pn;

497  if (!is\_constant\_struct<T\_scale>::value)

498  operands\_and\_partials.d\_x3[n]

499  -= - y\_inv\_dbl \* gamma\_p\_derivative(alpha\_dbl,

500  beta\_dbl \* y\_inv\_dbl) / Pn;

501  }

502

503  return operands\_and\_partials.to\_var(P);

504  }

505

506  template <class RNG>

507  inline double

508  inv\_gamma\_rng(const double alpha,

509  const double beta,

510  RNG& rng) {

511  using boost::variate\_generator;

512  using boost::random::gamma\_distribution;

513

514  static const char\* function = "stan::prob::inv\_gamma\_rng(%1%)";

515

516  using stan::math::check\_positive\_finite;

517

518  check\_positive\_finite(function, alpha, "Shape parameter", (double\*)0);

519  check\_positive\_finite(function, beta, "Scale parameter", (double\*)0);

520

521  variate\_generator<RNG&, gamma\_distribution<> >

522  gamma\_rng(rng, gamma\_distribution<>(alpha, 1 / beta));

523  return 1 / gamma\_rng();

524  }

525  }

526 }

527

528 #endif

stan::prob::inv\_gamma\_cdf\_log

return\_type< T\_y, T\_shape, T\_scale >::type inv\_gamma\_cdf\_log(const T\_y &y, const T\_shape &alpha, const T\_scale &beta)

**Definition:** inv\_gamma.hpp:294

stan::is\_constant

Metaprogramming struct to detect whether a given type is constant in the mathematical sense (not the ...

**Definition:** traits.hpp:43

multiply\_log.hpp

traits.hpp

stan::agrad::tgamma

fvar< T > tgamma(const fvar< T > &x)

**Definition:** tgamma.hpp:15

stan::is\_vector::type

T type

**Definition:** traits.hpp:85

stan::agrad::OperandsAndPartials::to\_var

T\_return\_type to\_var(double logp)

**Definition:** partials\_vari.hpp:138

stan::math::check\_positive\_finite

bool check\_positive\_finite(const char \*function, const T\_y &y, const char \*name, T\_result \*result)

**Definition:** check\_positive\_finite.hpp:12

error\_handling.hpp

stan::math::multiply\_log

boost::math::tools::promote\_args< T\_a, T\_b >::type multiply\_log(const T\_a a, const T\_b b)

Calculated the value of the first argument times log of the second argument while behaving properly w...

**Definition:** multiply\_log.hpp:51

stan::length

size\_t length(const T &)

**Definition:** traits.hpp:159

stan::DoubleVectorView

DoubleVectorView allocates double values to be used as intermediate values.

**Definition:** traits.hpp:358

partials\_vari.hpp

stan::agrad::value\_of

T value\_of(const fvar< T > &v)

Return the value of the specified variable.

**Definition:** value\_of.hpp:16

constants.hpp

stan::prob::inv\_gamma\_cdf

return\_type< T\_y, T\_shape, T\_scale >::type inv\_gamma\_cdf(const T\_y &y, const T\_shape &alpha, const T\_scale &beta)

The CDF of an inverse gamma density for y with the specified shape and scale parameters.

**Definition:** inv\_gamma.hpp:177

stan::is\_vector

**Definition:** traits.hpp:83

stan::agrad::lgamma

fvar< T > lgamma(const fvar< T > &x)

**Definition:** lgamma.hpp:15

stan::agrad::OperandsAndPartials

A variable implementation that stores operands and derivatives with respect to the variable...

**Definition:** partials\_vari.hpp:76

traits.hpp

stan::return\_type::type

boost::math::tools::promote\_args< typename scalar\_type< T1 >::type, typename scalar\_type< T2 >::type, typename scalar\_type< T3 >::type, typename scalar\_type< T4 >::type, typename scalar\_type< T5 >::type, typename scalar\_type< T6 >::type >::type type

**Definition:** traits.hpp:406

stan::is\_constant\_struct

Metaprogram to determine if a type has a base scalar type that can be assigned to type double...

**Definition:** traits.hpp:57

stan::math::value\_of

double value\_of(const T x)

Return the value of the specified scalar argument converted to a double value.

**Definition:** value\_of.hpp:24

stan::prob::include\_summand

Template metaprogram to calculate whether a summand needs to be included in a proportional (log) prob...

**Definition:** traits.hpp:35

stan::prob::inv\_gamma\_log

return\_type< T\_y, T\_shape, T\_scale >::type inv\_gamma\_log(const T\_y &y, const T\_shape &alpha, const T\_scale &beta)

The log of an inverse gamma density for y with the specified shape and scale parameters.

**Definition:** inv\_gamma.hpp:40

stan::agrad::gamma\_q

fvar< T > gamma\_q(const fvar< T > &x1, const fvar< T > &x2)

**Definition:** gamma\_q.hpp:15

stan::agrad::OperandsAndPartials::d\_x2

VectorView< double \*, is\_vector< T2 >::value, is\_constant\_struct< T2 >::value > d\_x2

**Definition:** partials\_vari.hpp:83

stan::math::gradRegIncGamma

double gradRegIncGamma(double a, double z, double g, double dig, double precision=1e-6)

**Definition:** internal\_math.hpp:181

stan::math::check\_nonnegative

bool check\_nonnegative(const char \*function, const T\_y &y, const char \*name, T\_result \*result)

**Definition:** check\_nonnegative.hpp:52

stan::prob::inv\_gamma\_rng

double inv\_gamma\_rng(const double alpha, const double beta, RNG &rng)

**Definition:** inv\_gamma.hpp:508

stan::math::check\_consistent\_sizes

bool check\_consistent\_sizes(const char \*function, const T1 &x1, const T2 &x2, const char \*name1, const char \*name2, T\_result \*result)

**Definition:** check\_consistent\_sizes.hpp:13

stan::max\_size

size\_t max\_size(const T1 &x1, const T2 &x2)

**Definition:** traits.hpp:191

stan::prob::inv\_gamma\_ccdf\_log

return\_type< T\_y, T\_shape, T\_scale >::type inv\_gamma\_ccdf\_log(const T\_y &y, const T\_shape &alpha, const T\_scale &beta)

**Definition:** inv\_gamma.hpp:401

internal\_math.hpp

stan::math::check\_less\_or\_equal

bool check\_less\_or\_equal(const char \*function, const T\_y &y, const T\_high &high, const char \*name, T\_result \*result)

**Definition:** check\_less\_or\_equal.hpp:52

stan::math::check\_not\_nan

bool check\_not\_nan(const char \*function, const T\_y &y, const char \*name, T\_result \*result)

Checks if the variable y is nan.

**Definition:** check\_not\_nan.hpp:56

stan::agrad::digamma

fvar< T > digamma(const fvar< T > &x)

**Definition:** digamma.hpp:16

value\_of.hpp

stan::agrad::OperandsAndPartials::d\_x1

VectorView< double \*, is\_vector< T1 >::value, is\_constant\_struct< T1 >::value > d\_x1

**Definition:** partials\_vari.hpp:82

stan::agrad::OperandsAndPartials::d\_x3

VectorView< double \*, is\_vector< T3 >::value, is\_constant\_struct< T3 >::value > d\_x3

**Definition:** partials\_vari.hpp:84

constants.hpp

stan::agrad::log

fvar< T > log(const fvar< T > &x)

**Definition:** log.hpp:15

stan::VectorView

VectorView is a template metaprogram that takes its argument and allows it to be used like a vector...

**Definition:** traits.hpp:275

stan::prob::gamma\_rng

double gamma\_rng(const double alpha, const double beta, RNG &rng)

**Definition:** gamma.hpp:491

stan::math::check\_greater\_or\_equal

bool check\_greater\_or\_equal(const char \*function, const T\_y &y, const T\_low &low, const char \*name, T\_result \*result)

**Definition:** check\_greater\_or\_equal.hpp:58

stan::math::negative\_infinity

double negative\_infinity()

Return negative infinity.

**Definition:** constants.hpp:123


---

[ Stan Home Page ]

*© 2011–2014,
Stan Development Team.*
